# Anterior nerves of the knee (ANK) block: A novel motor sparing fascial plane block for analgesia of the knee: Observational cohort study

**DOI:** 10.1097/MD.0000000000046035

**Published:** 2026-05-12

**Authors:** Onur Balaban, Ridvan Işik, Ali Eman, Keziban Karacan, Kemal Nas

**Affiliations:** a Faculty of Medicine, Department of Anesthesiology and Reanimation, Sakarya University, Sakarya, Turkey; b Division of Pain Medicine, Department of Physical Medicine and Rehabilitation, Sakarya University Training and Research Hospital, Sakarya, Turkey; c Department of Anesthesiology and Reanimation, Sakarya University Training and Research Hospital, Sakarya, Turkey; d Faculty of Medicine, Department of Anatomy, Sakarya University, Sakarya, Turkey; e Faculty of Medicine, Division of Pain Medicine, Department of Physical Medicine and Rehabilitation, Sakarya University, Sakarya, Turkey.

**Keywords:** anterior knee pain, fascial plane block, knee osteoarthritis, nerve block

## Abstract

Knee osteoarthritis is a widespread disorder that may contribute to severe chronic pain. Multiple nerve blocks are implemented for analgesia of the knee, which have some restrictions, including the need for multiple injections, making the procedure complicated. We aimed to introduce a novel ultrasound-guided nerve block technique for analgesia of the knee. The study presents a description of the anterior nerves of the knee block and its application to individuals with knee osteoarthritis. Local anesthetic was injected (20 mL of 0.25% bupivacaine) within the fascia between the rectus femoris and vastus muscles in 42 patients with severe knee pain. The pain charts of the patients were reviewed and numerical rating pain scores (NRS) before and 1st hour, 1st month after the block were evaluated. First-month Western Ontario and McMaster Universities Arthritis Index (WOMAC) was also reviewed. The block was also performed in a fresh embalmed cadaver with methylene blue to identify the distribution of the injected dye. The mean age of patients was 65.3 ± 7.8 years. The mean NRS before block performance was 8.0 ± 1.2. The NRS score at 1 hour and 1 month after the block, decreased to 1.5 ± 1 and 4.4 ± 1.2, respectively. The decrease was statistically and clinically significant (*P* = .000). Mean WOMAC score was 71.6 ± 14.5 before the block which decreased to 42.7 ± 14.9 one month after the block (*P* = .000). No muscle weakness, motor block, or block-related complications were observed. Methylene blue spread in the cadaver was between the rectus femoris and vastus muscles, covering the nerves that innervate the anterior region of the knee. The novel anterior nerves of the knee block provided sufficient analgesia at the first hour and first month, and improved 1-month WOMAC scores in patients with chronic pain at the anterior of knee.

## 1. Introduction

Knee osteoarthritis (KOA) is a widespread and debilitating musculoskeletal disorder that is particularly prevalent among older individuals, impacting around 3.8% of the global population.^[1,2]^ Given the growing number of elderly individuals and the rising prevalence of obesity, it is expected that the occurrence of knee osteoarthritis will substantially rise, leading to a significant economic burden on society.^[3]^

Various treatment modalities are available for KOA, encompassing conservative treatments including nonsteroidal anti-inflammatory drugs and physical therapy, as well as intra-articular or peri-articular injection treatments such as intra-articular steroids, orthobiologics, and surgical procedures.^[4,5]^ The Kellgren and Lawrence radiological classification indicates that a mild KOA consists of definite osteophytes and possible joint space narrowing, and moderate KOA is defined as presence of multiple osteophytes, definite narrowing of joint space, some sclerosis and possible deformity of bone ends.^[6]^ While conservative and injection therapies are successful in treating mild and moderate KOA, they are inadequate for the severe stage of KOA.^[6,7]^ Therefore, the surgical option becomes the most prominent choice. However, surgical interventions require a greater financial burden, and there are some patients with persistent pain and disability even after undergoing surgery.^[8,9]^ Hence, genicular nerve block and radiofrequency ablation are considered a feasible alternative to conservative treatment and surgical procedures such as total knee arthroplasty.^[10,11]^

**Figure 1.**
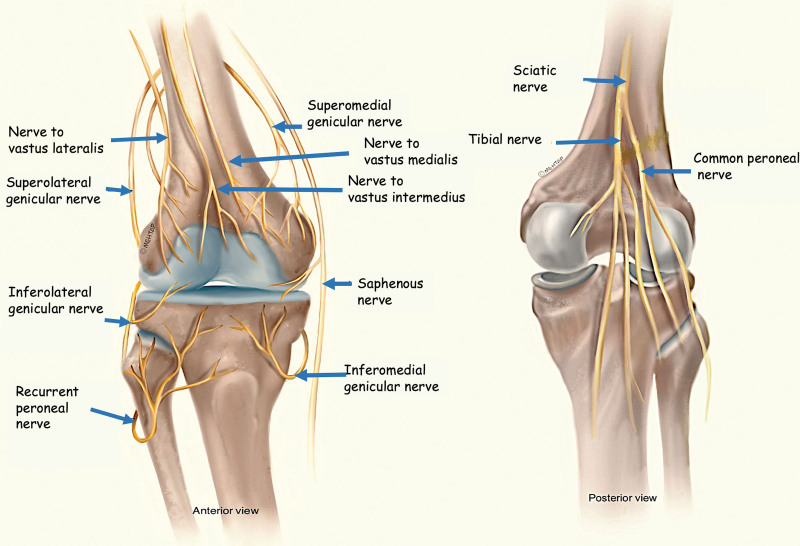
Illustration of complex innervation of the knee.

The knee joint has a complex, extensive, and wide range of innervation (Fig. 1). The articular branches emerging from the femoral, sciatic, and obturator nerves innervate the anterior capsule, though the obturator and sciatic nerves provide innervation to the posterior capsule.^[12]^ “Fascia lata,” the widest and thickest fascia of the body, is the part of the fascia profunda that covers the femur. The fascia lata consists of the septa, known as septum intermusculares, which forms compartments containing muscles, nerves, and vessels. The extensions of Fascia lata surround the other parts of Quadriceps femoris and Rectus femoris in the anterior compartment.

Thus, potential compartments are formed between the parts of the quadriceps femoris, where the articular branches innervate the anterior knee capsule.^[13,14]^ Tran et al demonstrated in their comprehensive anatomical investigation on cadavers that the nerves responsible for sensory innervation of the anterior knee predominantly traverse beneath the rectus femoris muscle within the fascia between the vastus muscles.^[15]^

Typically employed knee nerve block or ablation treatments for anterior knee pain selectively focus on only 3 out of the nerves that provide innervation to the knee joint: the superior lateral, superior medial, and inferior medial genicular nerves.^[16]^ Therefore, because of the intricate and diverse innervation, conventional nerve block methods are unable to completely denervate it. Additionally, identifying the specific genicular nerves in the nerve block procedure can provide challenges in certain cases, such as individuals who are obese or have total knee arthroplasty. Moreover, it becomes necessary to administer 3 separate injections to successfully block the identified nerves. In response to the aforementioned issues, we developed a novel method to block the nerves that supply sensation to the anterior knee using a single-injection method.

Based on this information, we aimed to describe a new, safe and easily performed ultrasound-guided fascial plane block technique to block the articular branches of the anterior knee. The potentially targeted branches are: the articular branches of the femoral nerve which include the nerve to vastus intermedius and the infrapatellar branch of the saphenous nerve. Additionally, the genicular nerves that primarily innervate the knee joint capsule which are consisted of multiple branches that are: the superolateral genicular nerve, superomedial genicular nerve. These nerves innervate the hip joint and the anterior knee region and they contribute to the sensory innervation of the knee joint capsule.

The primary objective of this study was to demonstrate the pain-relieving effectiveness of the novel anterior nerves of the knee (ANK) block in patients suffering from chronic knee pain caused by osteoarthritis. Additionally, the study aimed to assess the distribution of the injected solution in a cadaver to identify the nerves affected in the knee.

## 2. Methods

The trial was a retrospective cohort study including the assessment of the injectate distribution in a fresh embalmed cadaver. Patients diagnosed with Kellgren and Lawrence grade 3 and 4 knee osteoarthritis were included in the study. Grade 4 KOA is defined as: radiologically large osteophytes, marked narrowing of joint space, severe sclerosis and definite deformity of bone ends.^[6]^

The block was administered to 42 patients. Exclusion criteria were patients with a prominent anatomical knee disorder, patients having a severe cardiac or pulmonary disease, and patients having a contraindication of the regional block procedure such as infection at block site or a coagulation disorder or allergy to the local anesthetic drugs.

The patients were informed about the injection method before the intervention, and written informed consent was obtained. The study was approved by the Sakarya University institutional Ethics Committee (E-71522473-050.04-340179-38) and was carried out in compliance with the principles of the Declaration of Helsinki.

A pain medicine specialist (RI) carried out the block procedure. The blocks were performed under ultrasound (US) guidance in all cases (HM70 EVO, Samsung Healthcare, Seoul, South Korea) with a linear US transducer (10–12 MHz). The procedures were performed in the special block procedure room. Standard monitoring, including electrocardiography, noninvasive blood pressure, and peripheral oxygen saturation, was employed, and an IV catheter was inserted before the blocks.

**Figure 2.**
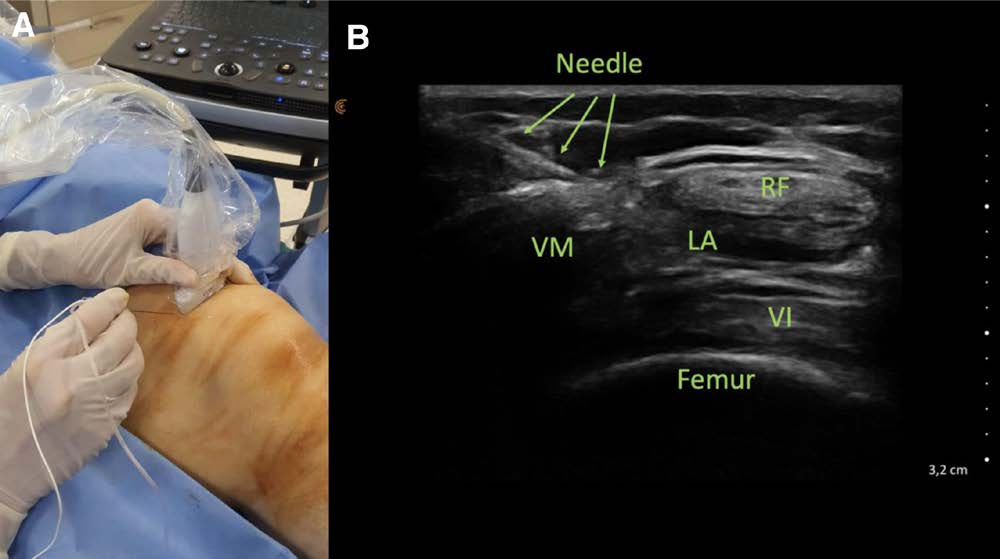
(A) The position of the ultrasound probe and the needle during the ANK block procedure. (B) The ultrasound image of the anatomical structures and the needle, and the distribution of the local anesthetic solution. ANK = anterior nerves of the knee.

The US probe was enclosed in a sterile cover, and then the patient’s skin was sterilized with iodopovidone. As the sonographic interface, a customized sterile gel was utilized. The patients were required to lie down in supine position, and subsequently, the ultrasound probe was initially placed in a transverse plane over the distal femur. In order to identify the rectus femoris muscle, we specifically targeted the upper anterior region of the knee. Once the rectus femoris muscle was identified, the transducer was advanced slightly medial (Fig. 2A). In this view, the fascia separating the vastus medialis and rectus femoris muscles is observed (Fig. 2B). A 22-gauge, 80-mm needle (Stimuplex A; BBraun, Melsung, Germany) was introduced medial to lateral direction using in-plane approach to place the tip in the fascial plane (Fig. 2B). After confirming negative aspiration, a single-injection block was conducted using 20 mL of 0.25% bupivacaine, observing adequate distribution of the fluid in this plane to complete the block procedure (Fig. 2B). The local anesthetic dose was determined based on the previous studies that implemented similar blocks performed for knee analgesia.^[17]^

After the procedure, we examined all patients for any muscle weakness using a manual muscle testing scale. This scale includes testing key muscles from the upper and lower extremities against the examiner’s resistance and grading the patient’s strength on a 0 to 5 scale accordingly. Post-block visits were done by the senior pain physician to observe the pain scores and to determine a possible motor weakness. Subjective pain measurements were performed during active movements. We reviewed the patients’ pain charts, including demographic data, operation information, and numeric rating scale (NRS) scores between 0 and 10 given by the patients (0 representing no pain and 10 representing the worst pain ever) before and after the procedures at the 1st hour and after 1 month. The Western Ontario and McMaster Universities Osteoarthritis Index (WOMAC) scores were also evaluated before and after the block procedures at the 1st hour and after 1 month (Table 1).

**Table 1:** Demographic and clinical characteristics of patients. The data is given as numbers, percentage, median (min–max), and mean ± standard deviation.

	Median (min–max)	Mean ± standard deviation
Age	64.0 (49.0–80.0)	65.3 ± 7.8
BMI (kg/m^2^)	31.5 (21.4–35.6)	31.3 ± 3.0
Symptom duration (month)	30.0 (6.0–80.0)	32.8 ± 18.2

		n (%)
KL grade	III	16 (38.1%)
	IV	26 (61.9%)
Gender	Female	31 (73.8%)
	Male	11 (26.2%)
Employment	Housewife	25 (59.5%)
	Government office	6 (14.3%)
	Labor	7 (16.7%)
	Retired	4 (9.5%)
Martial status	Married	34 (81.0%)
	Single	4 (9.5%)
	Divorced	4 (9.5%)

BMI = body mass index, KL = Kellgren–Lawrence scale for radiographic classification of osteoarthritis

We have conducted a cadaver study to reveal the distribution of injectate and describe the anatomic basis of the provided analgesia. We injected methylene blue dye at the described fascial plane of both lower extremity in a fresh embalmed cadaver. The dye was injected as 20 mL (1 mL of methylene blue diluted in 19 mL of saline). A brief cadaveric dissection was performed by our anatomy department to determine the spread of the injectate at different anatomic locations and to reveal the affected nerves. The cadaveric dissections were performed on the same day after the blocks.

### 2.1. Statistical analysis

The adequate number of patients to include in the study was assessed by using power analysis program (G*Power software, Heinrich-Heine-Universität, Düsseldorf, Germany). Based on the pilot study, in order to decrease pain from 5 to 3 (in a 10-scale pain chart) with a standard deviation of ±3 and an effect size of 0.66, alpha (α) error of 0.05 and beta (β) error of 0.95; sample size was calculated as 33 patients. Due to missing of data and refusal of participation, 42 patients were included in the study. Data were analyzed by using SPSS 28.0 (SPSS Inc., Chicago, IL) package program. Descriptive values were presented as number, frequency, mean ± standard deviation and median (minimum, maximum). The distribution of variables was measured using the Kolmogorov–Smirnov test. The Wilcoxon test was used to analyze dependent quantitative data. *P* < .05 was considered significant.

## 3. Results

The mean age of all patients was 65.3 ± 7.8 years. Of the patients, 31 (73.8%) were female and 11 (26.2%) were male. The mean duration of preprocedural pain in our patients was 32.8 ± 18.2 months. The mean body mass index of the patients was 31.3 ± 3.0 kg/m^2^. The demographic and clinical characteristics of the patients are given in Table 1.

**Figure 3.**
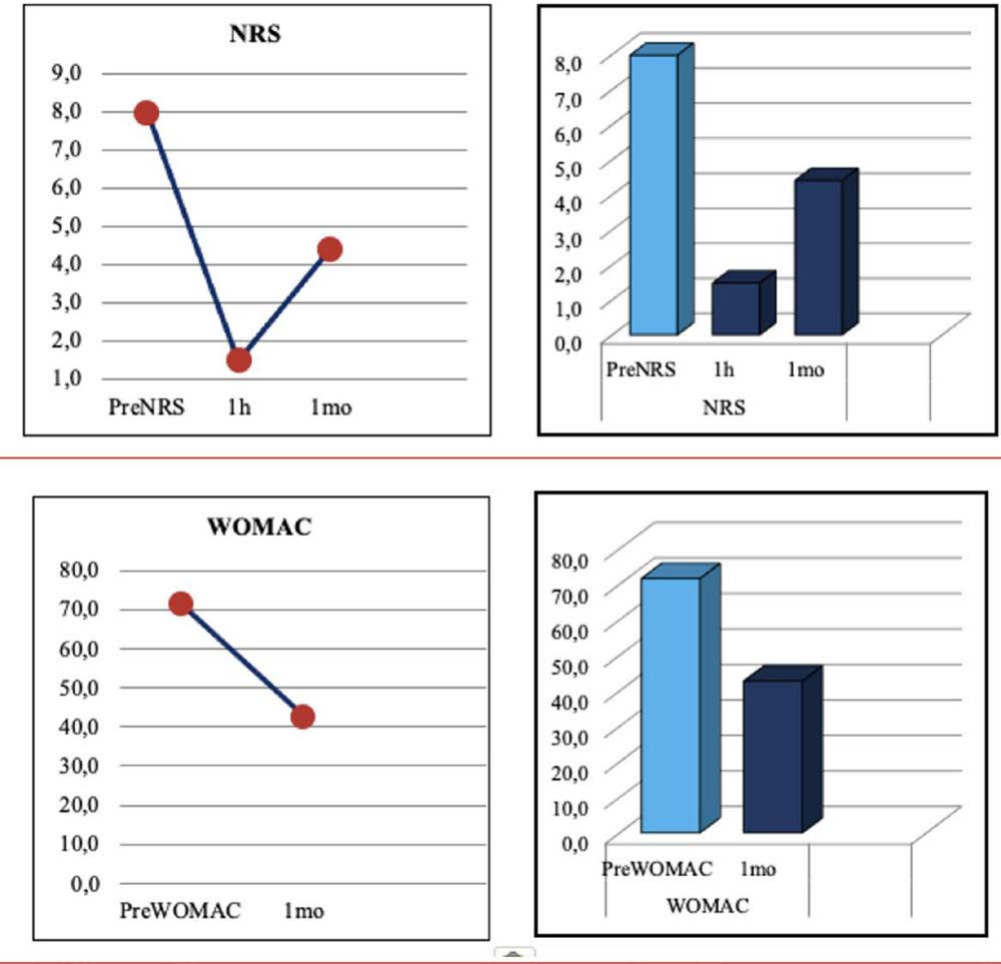
Changes of the pain and WOMAC scores in the patients at different time points. 1 h = first hour, 1 mo = first month, preNRS = numeric rating scale score before block, preWOMAC = WOMAC score before block.

All blocks were successful, and all patients declared a clinically meaningful decrease in pain levels as compared to their baseline (Fig. 3). The mean NRS pain score before block performance was 8 ± 1.2. The mean NRS score significantly decreased to 1.5 ± 1.1 one hour after the blocks (*P* = .000). The mean NRS score was 4.4 ± 1.2 one month after the blocks, the difference was found significant as compared to the pre-block mean NRS score (*P* = .000). The mean WOMAC score was 71.6 ± 14.5 before the blocks and significantly decreased to 42.7 ± 14.9 after 1 month (*P* = .000). The pain scores and WOMAC values of individual patients before the blocks, at 1st hour, and 1st month were given in Table 2.

**Table 2:** Mean ± standard deviation and median (minimum–maximum) values of pain scores and WOMAC index before and after the blocks.

	Pre-block	1st hour	1st month	*P* value
NRS				
Mean ± SD	8.0 ± 1.2	1.5 ± 1.1	4.4 ± 1.2	.000
Median (min–max)	8.0 (6.0–10.0)	1.0 (0.0–4.0)	4.0 (2.0–8.0)	.000
WOMAC				
Mean ± SD	71.6 ± 14.5		42.7 ± 14.9	.000
Median (min–max)	77.0 (39.0–89.0)		43.0 (20.0–72.0)	.000

NRS = numeric rating scale, WOMAC = The Western Ontario and McMaster Universities Osteoarthritis Index.

During each post-block visit, the patient’s muscle strength was assessed manually, their gait was evaluated, and the injection site was checked. No block-related complications such as muscle weakness, motor block, gait deterioration or site infection were observed in our patients.

**Figure 4.**
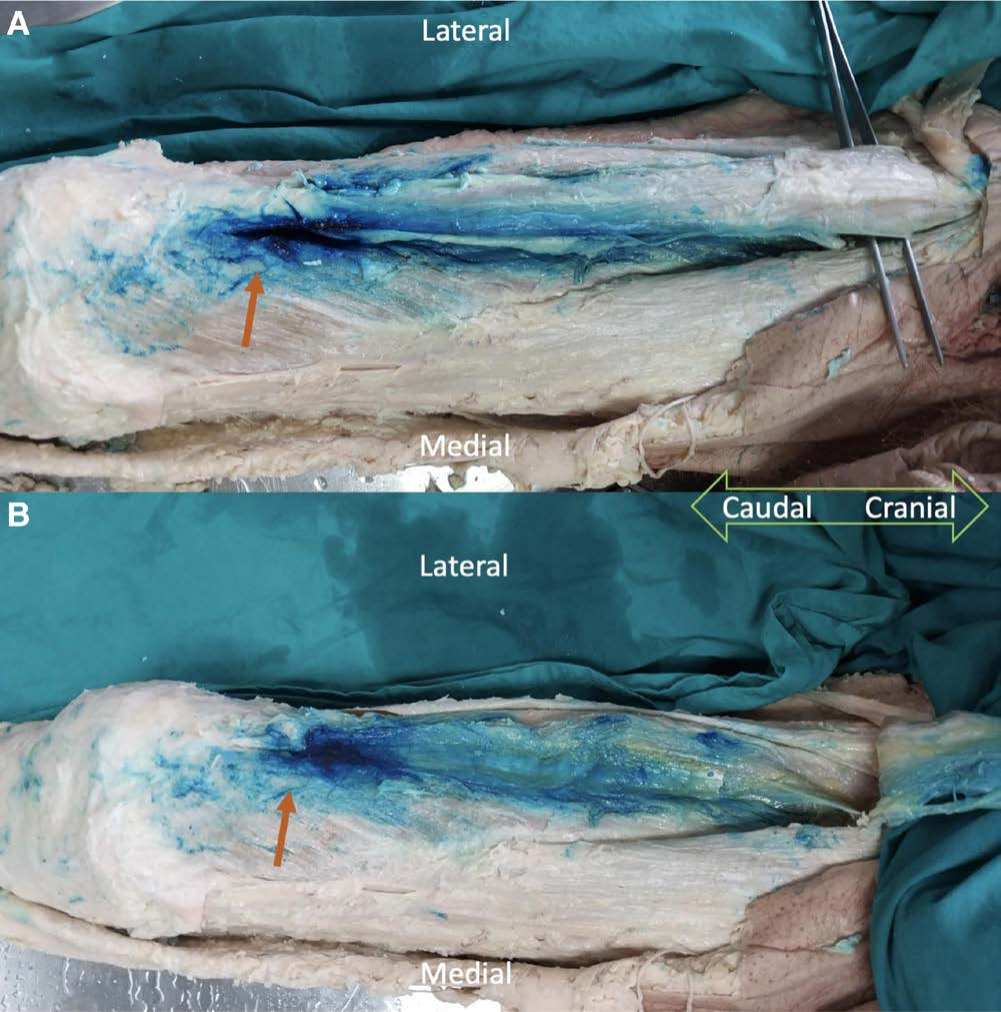
Spread of dye injected posterior to the rectus femoris muscle in the cadaver. (A) The rectus femoris muscle is dissected and lifted up. The dye is distributed along the rectus muscle posteriorly, also reaches to the edges of the muscle. (B) The rectus femoris muscle is removed and the distribution of the dye anterior to vastus muscles is seen. The dye spreads widely along the vastus muscles which reaches to the anterior branches of the obturator nerve.

**Figure 5.**
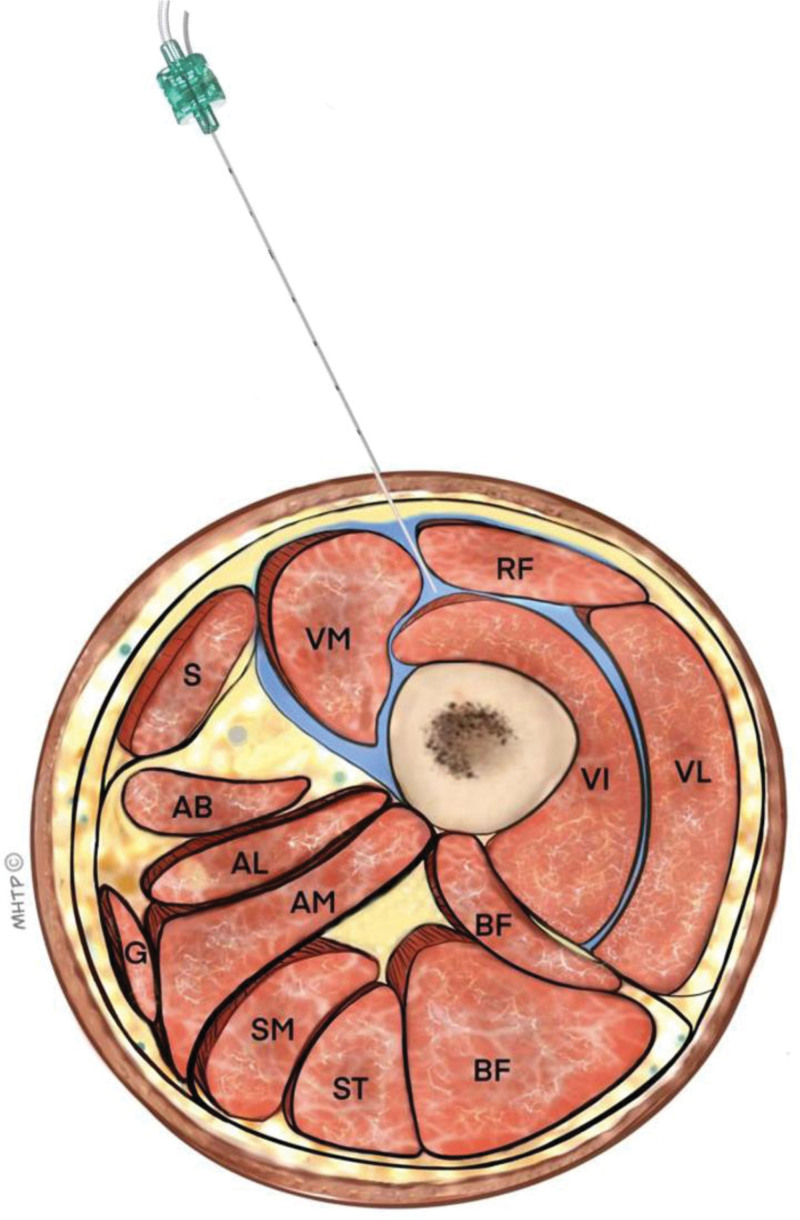
Illustration of the injected dye between the rectus femoris and vastus muscles, shown in transverse section. AB = adductor brevis, AL = adductor longus, AM = adductor magnus, BF = biceps femoris, G = gracilis muscle, RF = rectus femoris, S = sartorius, SM = semimembranosus, ST = semitendinosus, VI = vastus intermedius, VL = vastus lateralis, VM = vastus medialis.

**Figure 6.**
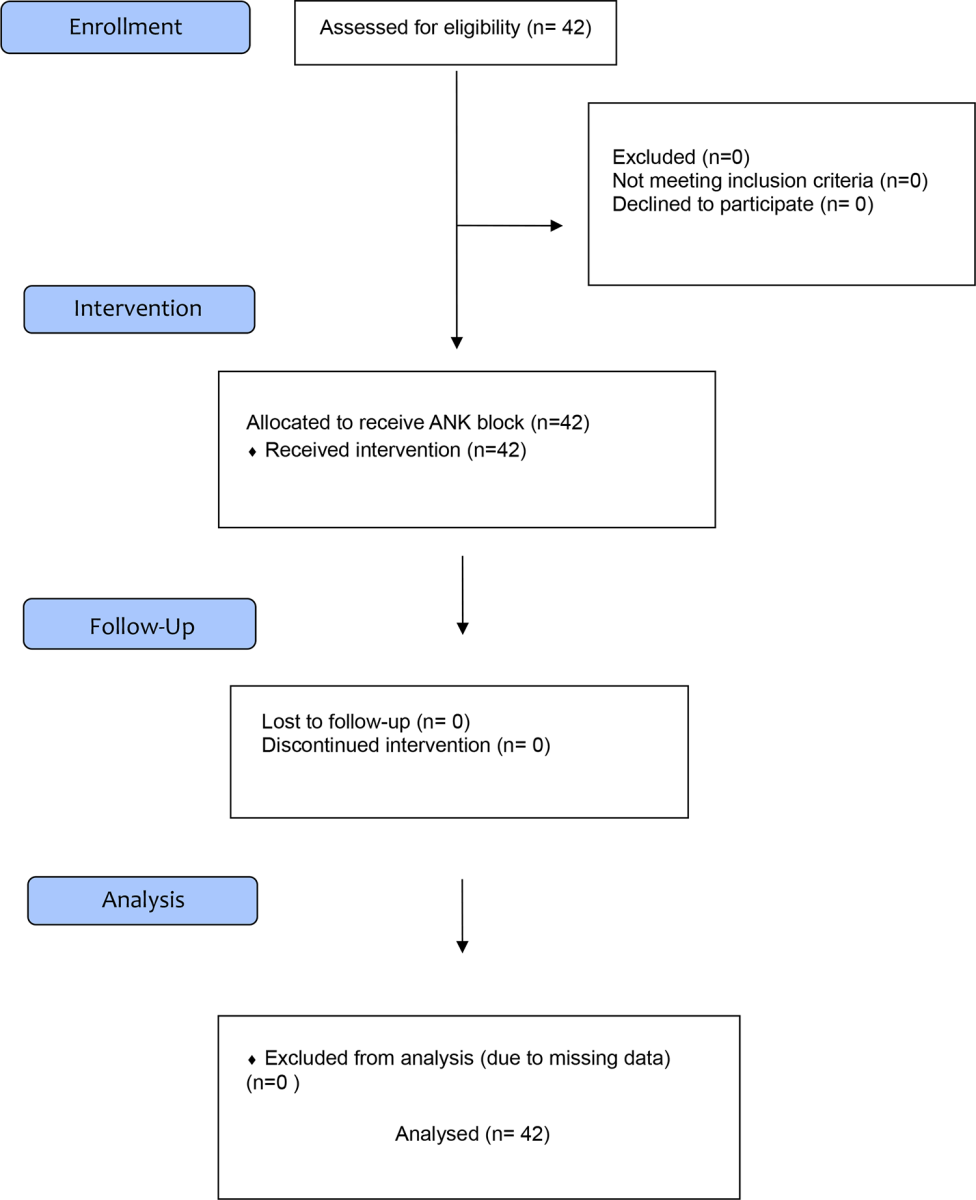
The flow-diagram of the study.

Methylene blue used in the cadaver was observed to diffusely spread both medially and laterally between the rectus femoris and vastus muscles in the anterior compartment of the knee (Fig. 4). The covering nerves are detected as: the nerve to the vastus medialis, the nerve to the vastus intermediate, and the nerve to the vastus intermediate, and the nerve to the vastus lateralis. The spread of the dye also reached the lateral and medial corners of the knee, which may affect the terminal branches of the superolateral and superomedial genicular nerves. However, despite the spread between the vastus muscles and adductor muscles, there was no spread to the adductor canal, where the saphenous nerve is located. The distribution of the dye in the transverse plane is illustrated in Figure 5. The flow-diagram of the study is shown in Figure 6.

## 4. Discussion

The primary study of the ANK block demonstrated that the ANK block provides effective analgesia, and showed promising results for managing acute short-term pain in patients with gonarthrosis. Compared to the other alternative knee analgesia methods, the ANK block has the advantage of being an easy-to-apply method that is performed by a single-injection with ultrasound guidance. Adductor canal block was offered for analgesia of the knee after knee arthroplasty.^[18]^ However, this block covers the medial part of the knee and may not be sufficient enough as a single method which requires additional blocks in combination. The combination of adductor canal block and interspace between the popliteal artery and capsule of the posterior knee block is more effective method, though it is not easy to perform.^[18]^ Epidural analgesia is an effective and sophisticated method for knee pain relief however, epidural catheter placement needs special training and a deeper injection with higher risk of complications.^[19]^ Femoral nerve block and lumbar plexus blocks have the disadvantage of possibly causing motor weakness which are not recommended any more in the context of multimodal analgesia.^[19]^

The ANK block covers a larger analgesia area of the anterior knee with a single injection of local anesthetic. In contrast, the alternatives to ANK block—such as genicular nerve block—require multiple injections. When combined with prolongation methods such as placing a catheter or the use of long-acting drugs, the ANK block can offer long-term pain relief for chronic pain. The main disadvantages of the ANK block are the coverage area of the block confined to the anterior portion of the joint and the anterior dermatomes of the knee. The denervation of the posterior region of the knee may require further interventions.

The sensory innervation of the knee is known to have a high degree of anatomical variability, and its complexity has been mentioned in recent anatomy studies.^[7,20,21]^ Thus, analgesia of the knee usually needs individualized methods utilizing more than 1 injection to cover a larger area of analgesia. A recent comprehensive cadaver study has traced the branches of 3 lower limb nerves to their termination and has marked 5 different articular branches that are potential targets for interventional procedures.^[21]^ Injection of a fascial plane to reach all these branches of the main lower limb nerves by performing a single-injection method seems feasible and effective. Although an excessive number of different fascial plane blocks have been introduced for injection at many different anatomic locations, thus far, a specific fascial plane has not been described, especially targeting the nerves of the knee. The innervation of the anterior region of the knee arises primarily from the femoral, obturator, and sciatic nerves. The articular branch of these nerves pass the rectus femoris muscle posteriorly in the plane between the vastus muscles and rectus femoris muscle. An interfascial plane injection between these muscles could possibly cover the nerves to vastus lateralis, vastus medalis and vastus intermediate.

The injectate in the cadaver also reached the anterior branch of the obturator nerve, which passes over the surface of the adductor brevis muscle. Upon analyzing the distribution of methylene blue on the cadaver, it was observed that the facial plane injection effectively covers the regions through which the neurons responsible for transmitting anterior knee sensation traverse. Also, the nerves arising from the anterior branch of the obturator nerve, which passes anteriorly to the adductor muscles, seem to be affected. However, the saphenous nerve is located in a separate fascial plane and was not dyed.

The block was found effective, especially in patients having knee pain at anterior region with analgesia in active movements. The ANK block may provide adequate analgesia in the operated patients as well, as a postoperative analgesia method. Postoperative analgesia in knee operations usually requires combination of multiple blocks.^[22]^ A single shot ANK block may cover the knee joint as well as the incision area. Thus, the analgesia area of the ANK block may be adequate for middle anterior incisions in knee arthroplasty operations.

Our research has limitations due to the small number of patients and the short-term evaluation, which may impact the results. However, this article is the primary description of a novel method, and we mainly focused on identifying the block technique. Another limitation is the observational, non-comparative character, which lacks a control group. The effectiveness of the ANK block is still a topic of further research in different patient populations, such as those having acute postoperative knee pain and trauma patients. Additionally, the duration of analgesia could be extended with the use of corticosteroids, adjuvant drugs, or by placing a catheter, all of which warrant future research. Randomized controlled studies are also essential to compare this method to alternative blocks and with traditional analgesic methods for the knee. The potential complications include muscle weakness, motor block, gait deterioration, local anesthetic toxicity or a site infection. Block related complications were not observed in our cohort. Motor weakness is a major problem for the blocks implemented in knee analgesia however, in our patients this was not present. The ANK block is likely to be a motor sparing block as it did not affect the motor nerves. Furthermore, a larger cohort of patients is needed to assess block related complications.

## 5. Conclusion

The novel ANK block is a promising method that offers significant pain relief in the anterior region of the knee. However, further studies are needed to investigate the effectiveness of the ANK block in a more diverse and large number of patients. Future research should also investigate the feasibility of prolonging the analgesia duration of this method and compare it with conventional treatments and other alternative approaches.

## Acknowledgments

The authors thank Dr Mehtap Erdoğan from Anatomy Department of Sakarya University Medical Faculty for her valuably assistance and for the illustrations.

## Author contributions

**Conceptualization**: Onur Balaban, Ridvan Işik.

**Data curation**: Onur Balaban, Keziban Karacan.

**Formal analysis**: Onur Balaban, Ridvan Işik, Ali Eman, Keziban Karacan.

**Investigation**: Onur Balaban, Ridvan Işik.

**Methodology**: Onur Balaban.

**Resources**: Ali Eman.

**Supervision**: Kemal Nas.

**Writing – original draft**: Onur Balaban, Ridvan Işik.

**Writing – review & editing**: Onur Balaban, Ridvan Işik, Ali Eman, Keziban Karacan, Kemal Nas.
